# Systematic Comparison of Homogeneous Catalyst Recycling Strategies: Organic Solvent Nanofiltration vs. Liquid‐Liquid‐Multiphase

**DOI:** 10.1002/chem.202503075

**Published:** 2025-12-24

**Authors:** Sven Störtte, Lisa Steinwachs, Rucha S. Medhekar, Robinson Novemen, Andreas J. Vorholt

**Affiliations:** ^1^ Max Planck Institute For Chemical Energy Conversion Mülheim an der Ruhr Germany

**Keywords:** comparison, homogeneous catalysis, Multiphase, Organic Solvent Nanofiltration, recycling strategies

## Abstract

This study presents a systematic comparison of two recycling strategies for molecular catalyst—multiphasic catalyst recycling and organic solvent nanofiltration (OSN)—within a unified reaction system. Molecular catalyzed reactions are vital in chemical production, necessitating efficient recycling of costly catalyst metals. To facilitate a fair comparison, a versatile model reaction system was developed that allows for both monophasic and multiphasic configurations while preserving the integrity of the central hydroformylation process, converting 1‐hexene and CO/H_2_ syngas to heptanal in both cases.

## Graphical abstract

Key performance metrics, including hexene conversion rates and catalyst leaching, were evaluated over extended continuous operation periods. Results demonstrated that the OSN method significantly outperformed the multiphasic system, maintaining hexene conversion at 83% after 76 h compared to a decline to 72% in the multiphasic setup after 36 h. Furthermore, catalyst leaching was substantially lower in the OSN system, at below 0.1 ppm during continuous operation an order of magnitude less than the competing multiphasic approach.

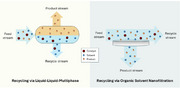

## 1 Introduction

Molecular catalyzed reactions nowadays account for millions of tons of chemical products annually and are therefore an integral part in the chemical production network. For being economically viable, these usually involve recycling of the often‐expensive catalyst metal, which therefore needs to be separated from the product stream [1, 2]. Modelling comparing chemical separation technologies for energy saving potential has been explored recently [3]. Efficient separation of products from catalysts can be achieved through various methods based on volatility differences, including stripping, distillation, and rectification [4]. Most molecular catalysts are nonvolatile, allowing for thermal removal of products while the catalyst remains in the reaction mixture [5]. Distillation is particularly favored in industrial applications, such as the Monsanto and Cativa processes for acetic acid production, where volatiles are effectively removed in a flash‐type distillation setup [6, 7]. Additionally, hydroformylation processes involving cobalt‐carbonyls [8, 9] or the Reppe process for propionic acid production [10] demonstrate the versatility of distillation in recycling homogeneous catalysts.

Another method involves exploiting partitioning coefficients between two liquid phases, as seen in the Kuraray process for 2,7‐octadienol production, where a water‐soluble catalyst is retained in an aqueous phase while the nonpolar product is extracted using hexane [11]. This principle is also applied in liquid/liquid biphasic catalysis, which allows for direct decantation of products. The Shell Higher Olefin Process (SHOP) exemplifies this approach, producing 1‐alkenes from ethylene, where the low‐polarity products form a separate liquid phase [12]. Similarly, the Ruhrchemie/Rhône‐Poulenc process for hydroformylation of short‐chain olefins like propylene employs a rhodium catalyst immobilized in water by sulfonated ligands, allowing for efficient separation of butanal from the aqueous phase [13, 14].

Separation can also be achieved through solubility differences, where precipitation of either the catalyst or product occurs [15, 16, 17, 18]. The use of supercritical CO_2_ to tune solubility properties has been explored in academic settings, providing a novel approach to catalyst recycling [19, 20, 21]. Additionally, the precipitation method has been applied for an entire solvent‐catalyst matrix, which was obtained after cooling below the melting point of the catalyst‐containing ethylene carbonate phase. The unpolar hydroformylation products remained liquid and could be decanted off [22].

Lastly, organic solvent nanofiltration (OSN) utilizes membranes to separate compounds based on size and polarity. Recent publications show ongoing progress in that field [23, 24, 25, 26]. Allowing smaller products to pass while retaining larger catalysts, this method is applied in processes by companies like Evonik [27, 28, 29], and is also academically explored in continuous processes [30, 31, 32, 33, 34].

The enlargement of ligand substituents is a common strategy in this approach, increasing the size and molecular weight of the catalyst to favor retention compared to the products [35, 36].

Nonetheless, literature lacks a systematic comparison of separation strategies for a single reaction system. Usually, the adoption of multiphasic recycling incorporates ligand modifications to favor its presence in the recycled phase, compared to ligands designed to stay in the product phase for OSN and distillation approaches. Therefore, the homogeneous catalyst system is changed, obscuring performance implications of the respective recycling strategy behind those of the altered catalyst. To compare inherent performance bearing of recycling strategies, the catalyst system needs to remain unchanged.

In this work, a versatile reaction mixture design allows for two recycling strategies that are otherwise difficult to realize without tuning ligands, to be compared in that manner:

1. multiphasic catalyst recycling, where the catalyst metal is bound to a recyclable polar phase by ligands With ionic moieties,
2. and recycling by organic solvent nanofiltration (OSN), with a membrane retaining the catalyst complexes inside the reaction system while products can pass the membrane.

**FIGURE 1:**
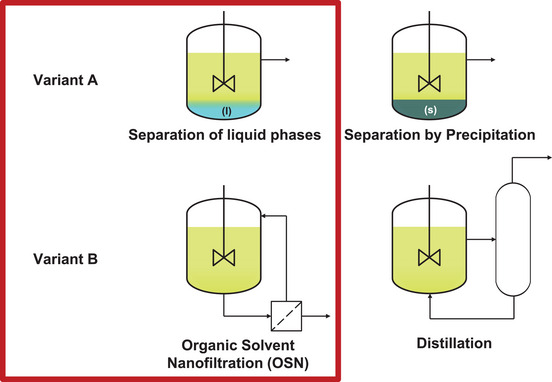
Different separation concepts in homogeneous catalysed processes and the variants compared in this work.

Figure 1 illustrates the general concept of both competing catalyst recycling strategies, highlighted as variant A and B. Both concepts are realized as continuous processes to observe their long‐term behavior.

## 2 Results and Discussion

The chosen model reaction hydroformylation produces aldehydes from alkenes. In usual applications of hydroformylation reaction systems reactant and product form a single organic phase matrix for the catalyst to be dissolved in [27]. Examples include hydroformylation of 1‐dodecene in toluene with Biphephos, Xantphos, and TPP ligands [30] and 1‐dodecene and 1‐octene in mixtures with their hydroformylation products, organic high boilers and bulky phosphite ligands [29]. To be able to compare both monophasic and multiphasic versions of the same reaction system, naturally, changes have to be made to achieve a second liquid phase to form. The reaction system was therefore chosen to keep the changes necessary as minuscle as possible and without expectable influence on the hydroformylation reaction from a chemical perspective. Differences in performance between the two catalyst recycling approaches were aimed for to be mostly attributable to consequences of said recycling strategies.

A tandem reaction system developed by our group exhibits those properties. Coupled to a hydroformylation of 1‐pentene, an aldol condensation of the product hexanal renders the reaction mixture biphasic. The Aldol condensation products form a second phase that demixes from the catalyst phase. Recyclability of the Rhodium/Sulfoxantphos hydroformylation catalyst as well as of the added basic catalyst for aldol condensation is achieved via a polar polyethyleneglycol‐200 (PEG‐200) phase which binds the catalysts by the ionic properties of an anorganic Brønstedt base and the ligand Sulfoxantphos [37]. The addition of a basic catalyst is the only change necessary to render the system biphasic from a previously monophasic hydroformylation mixture.

**FIGURE 2:**
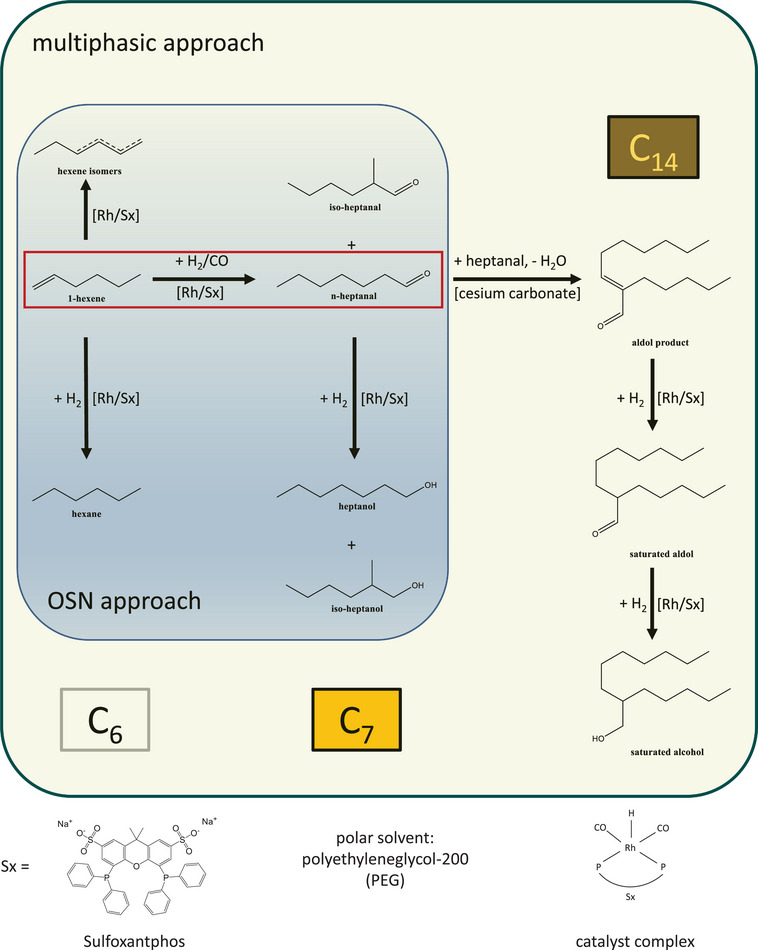
Reaction network of Hydroformylation (multiphase and OSN approach, marked in blue) and consecutive, Brønsted base‐catalyzed Aldol condensation (multiphase approach only), with respective side products likely to occur, starting from 1‐hexene as substrate.

For continuous operation, only slight adjustments had to be made to the literature system, nonetheless without changing the character of the reaction system. Due to the low boiling point of 1‐pentene, which is no issue for autoclave experiments but a detriment to continuous pumping of a feed stream at ambient conditions, a switch to 1‐hexene as a substrate was inevitable for practical reasons. The reaction system used for this work is shown in Figure 2.

**FIGURE 3:**
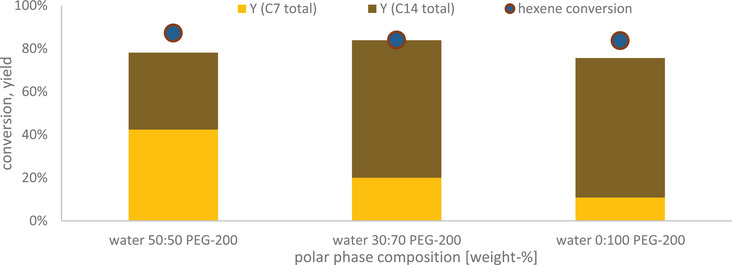
Hexene conversion, product yields and n/iso ratio for the multiphasic reaction system with different water accumulation scenarios. 50:50 corresponds to full accumulation of water by the product phase after 40 h of continuous operation. Reaction conditions: T = 125°C, p = 50 bar, c_0, Rh(acac)(CO)2_ = 1,44 g L^−1^, c_0, Sulfoxantphos_ = 8,77 g L^−1^, x_Rh/p_ = 1:4, CO/H2 = 1:1, 1‐hexene: 2.0 mL, polar phase: 0.8 mL, t = 5 h, stir rate = 1500 rpm Difference of hexene conversion and hydroformylation yield is hydrogenation byproduct hexane.

This reaction system presents a significant advantage for the comparability approach of this work: Only for the case of adding the basic catalyst cesium carbonate a second phase is formed at a conversion rate of 25% to the aldol product. Water as side product is not affecting the reactivity of the hydroformylation. Assuming continuous addition of produced water to the polar phase, the latter would be composed of 30% water after 15 h and 50% water after 40 h of continuous operation. Autoclave experiments show that even though the product composition changes in terms of C_14_ fraction due to weaken base strength [37], hexene conversion stays constant, proving hydroformylation to be independent in that regard (Figure 3). As a small molecule, water is additionally likely to be separated from the catalyst phase by continuous leaching to the product phase, resulting in a lesser extent of water accumulation in the actual process application.

This allows hydroformylation to proceed unaffected if no second catalyst is added in a polar, monophasic system (Figure 2, marked in blue). With catalyst recycling via OSN, this setup can be compared with the multiphasic approach under otherwise identical conditions. Table 1 displays average yields from both systems, confirming that hydroformylation is active in both monophasic and biphasic systems.

**TABLE 1:** Average yields of stable continuous process between 5 and 10 h into the continuous process

Table entry	Process variant	X (hexene)	Y (C_7_ products)	Y (C_14_ products)	Aldehyde/ alcohol ratio	Y (hydroformylation, total)
1	A (multiphase)	95%	40%	55%	8	95%
2	B (OSN)	95%	81%	12%	4	93%

Reaction conditions: T = 125°C, p = 50 bar, c_0, Rh(acac)(CO)2, reactor_ = 1,44 g L^−1^, c_0, Sulfoxantphos, reactor_ = 8,77 g L^−1^, x_Rh/p_ = 1:4, $\overset{.}{\underset{CO}{n}}\;\;$ = $\overset{.}{\underset{H2\;}{n}}\;$ = 1 mol h^−1^, $\overset{.}{\underset{Hexene}{V}}$ = 16 mL h^−1^, $\overset{.}{\underset{Recycle}{V}}$   =  200 mL h^−1^, $\;\;\overset{.}{\underset{Membrane\;crossflow}{m}}=$ 60 kg h^−1^ Shown are average yields (Y) and hexene conversions (X) in stable continuous conditions between 5 and 10 h after start of the continuous feed of 1‐hexene. Difference of hexene conversion and hydroformylation yield is hydrogenation byproduct hexane. C_14_ product yield in the OSN process is due to thermal activation at reaction temperature.

**FIGURE 4:**
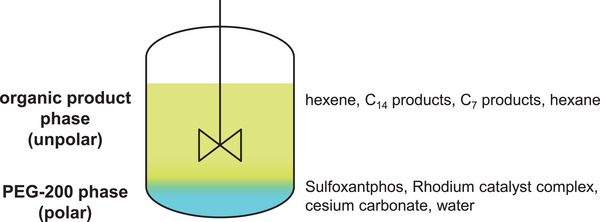
Distribution of products, reactants, and catalysts to the liquid phases in case of the multiphasic approach

This indicates that the reaction setups are suitable for comparison based on the advantages and disadvantages of their respective catalyst retention techniques during long‐term continuous operation. Table 2 lists the reaction conditions used for continuous operation of the two respective processes, Figure 4 shows the distribution of products, reactants and catalysts to the liquid phases in case of the multiphasic approach.

**TABLE 2:** Hydroformylation reaction conditions for both cases

Reaction parameters	
Pressure	50 bar
Reaction temperature	125°C
Stir rate	2000 rpm
CO/H_2_‐ratio	1:1
Catalyst precursor	Rh(acac)(CO)_2_
Ligand	Sulfoxantphos (Figure 2)
Ligand/metal ratio	2:1
Solvent	Polyethyleneglycol‐200 (PEG 200)
Solvent volume fraction in reactor	0.3
Substrate feed (1‐hexene)	16 mL h^−1^

**FIGURE 5:**
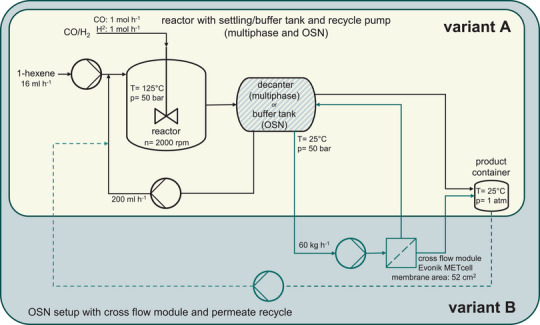
Continuous reaction setups for comparing OSN‐assisted and multiphasic catalyst recycling. A detailed description of the equipment can be found in the SI (Figure S2).

Figure 5 shows the continuous operated miniplant setups used for either recycling option. The two process setups share the same reactor but the downstream recycling system is configured differently to serve distinct functionalities. In the multiphasic system, a dip tube extracts excess liquid from the reactor, directing it to a decanter where two phases settle. The heavier phase is recycled back into the reactor, while the lighter product phase is sent to a product container. In contrast, the OSN‐assisted process also utilizes the dip tube for liquid extraction but repurposes the downstream tank as a buffer tank for a secondary membrane separation circuit, stabilizing liquid levels and maintaining concentration homogeneity between the buffer and reactor. The flat sheet membrane module operates in cross‐flow mode, with its permeate flow collected in the product container and excess permeate recycled to the reactor to ensure constant liquid levels. More detailed descriptions of both setups can be found in the Experimental Section.

### 2.1 Multiphasic Reaction Run

**FIGURE 6:**
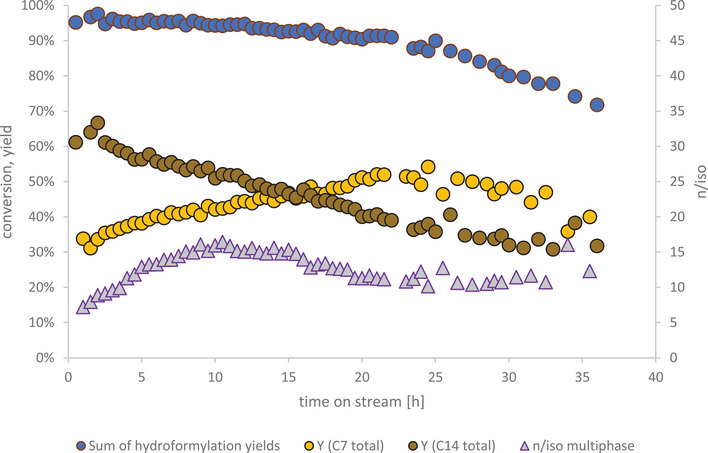
Hexene conversion, product yields and n/iso ratio for the multiphasic reaction system. Reaction conditions: T = 125°C, p = 50 bar, c_0, Rh(acac)(CO)2, reactor_ = 1,44 g L^−1^, c_0, Sulfoxantphos, reactor_ = 8,77 g L^−1^, c_0, cesium carbonate, reactor_ = 45,2 g L^−1^, x_Rh/p_ = 1:4, $\overset{.}{\underset{CO}{n}}\;\;$ = $\overset{.}{\underset{H2\;}{n}}\;$ = 1 mol h^−1^, $\overset{.}{\underset{Hexene}{V}}$ = 16 mL h^−1^, $\overset{.}{\underset{Recycle}{V}}$   =  200 mL h^−1^

The multiphasic reaction was conducted in variant A of the miniplant setup, using a decanter for phase separation. Figure 6 shows its results.

The continuous reaction ran for 36 h in total. After 22 h hexene conversion began dropping steeply from >90% to 72% within 14 h and the experiment was stopped. The aldol condensation decreased steeply as well, showing deactivation of the basic catalyst due to some accumulation of the side product water, as predicted [37]. As a diol, polyethylene glycol likely behaves like the comparable solvent 1,2‐ethanediol, in which weak bases exhibit higher basicity than in water [38], reducing their catalytic activity. With hydroformylation yields staying above 90% 22 h into the continuous experiment, decreasing aldol condensation reaction rates translated mostly into more heptanal being left in the reaction mixture, thus rising C_7_ yields. The observed n/iso ratio rises from 7.1 to 16.4 after startup as seen in other continuously operated hydroformylation processes at high conversions [39], and stays in a corridor between 10 and 16 for most of the experiment. At these high values expected for a bidentate ligand like Sulfoxantphos, fluctuations of iso‐heptanal production remain small. Throughout the experiment, iso‐heptanal makes up between 6 and 9% of the total heptanal produced. From the 22 h mark on, rapid decline in hydroformylation yields occurs and the experiment was stopped after 36 h.

### 2.2 Membrane Screening

To continuously run the monophasic reaction system a membrane ready to facilitate the desired catalyst recycling was needed. Therefore, five commercially available OSN membranes, chosen by their reported usability for large ligand retention in homogeneous catalysis, were tested with an artificially assembled reaction solution mimicking the expected composition in a continuously operated reaction run, corresponding to 80% hexene conversion in a mixture with a 0.3 volume fraction of PEG‐200, as used in the multiphasic system. The tests were conducted in a crossflow setup with permeate refeed for continuous testing under constant conditions, using *Evonik METcell* 52cm^2^ cells. The setup is detailed in the SI (Figure S3).

Since any Rhodium‐Sulfoxantphos complexes that form under reaction conditions are larger than the Sulfoxantphos molecule itself, retention of the catalyst complexes would be ensured if a membrane rejected Sulfoxantphos transfer across it. Therefore, Sulfoxantphos retention was tested with the available membranes. A transmembrane pressure of 50 bar was applied to emulate reaction conditions.

**FIGURE 7:**
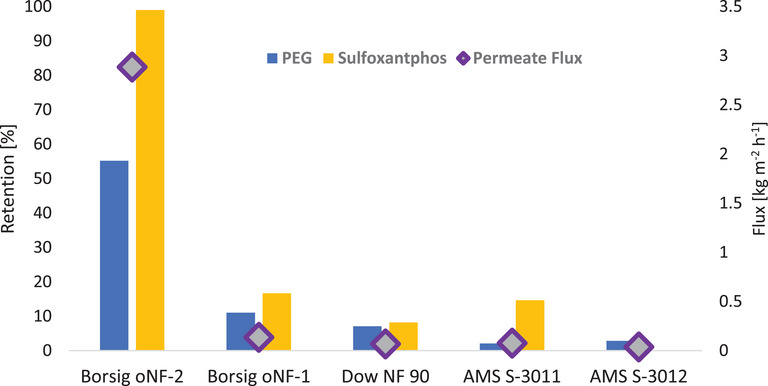
Sulfoxantphos and PEG retention and permeate flux of 5 commercially available membranes. Separation conditions: p = 50 bar, c_0, Sulfoxantphos_ = 8,77 g L^−1^, substrate‐product volume fraction = 0.7, PEG volume fraction = 0.3, hexene:heptanal ratio = 20:80, $\overset{.}{\underset{Membrane\;crossflow}{m}}=$ 60 kg h^−1^, A_membrane_ = 52 cm^2^

Figure 7 shows that of all tested membranes, only the *Borsig oNF‐2* membrane achieved significant retention of Sulfoxantphos. Next to 99% with oNF‐2, retention values of below 20% with *Borsig oNF‐1*, *DOW Filmtec NF 90*, *AMS S‐3011* and *AMS S‐3012*, which do not suffice for catalyst retention even in a multistage approach, were found unsuitable. Also, the permeate flux was considerably lower for all membranes compared to the *oNF‐2*, with 2.9 kg m^−2^ h^−1^. Possibly, the *oNF‐2* being cross‐linked to a higher degree than the *oNF‐1* originally designed for higher fluxes was less compacted by the relatively high transmembrane pressure of 50 bar, thus leaving pores permeable. The pores of the *oNF‐1* membrane, on the other hand, seem to have been compacted to allow only for a flux of 0.13 kg m^−2^ h^−1^. In case of the *Dow NF 90* a similar issue can be assumed, as it is commercially sold to be used under a maximum operating pressure of 41 bar. Both *AMS* membranes tested feature a nominal maximum operating pressure of 70 bar but were nonetheless found to permit almost no permate (0,08 kg m^−2^ h^−1^ with *AMS S‐3011*, 0,04 kg m^−2^ h^−1^ with *AMS S‐3012*). In case of the *AMS S‐3012* the flow was as low as to permit just enough sample within 4 h to perform GC analysis and calculate PEG retention, whereas the sample volume necessary for Sulfoxantphos quantification could not be achieved within a reasonable runtime.

Among all membranes tested, the *Borsig oNF‐2* outperformed the other membranes in terms of permeate flux and Sulfoxantphos retention by significant margins, mandating a clear choice pro *oNF‐2*. Additionally, the *oNF‐2* membrane retained 55% of PEG, exceeding PEG retention of the other tested membranes fivefold or more. Thus, the *oNF‐2* membrane significantly reduces demand for solvent refill during the continuous experiment as an additional feature.

### 2.3 OSN‐assisted Reaction Run

**FIGURE 8:**
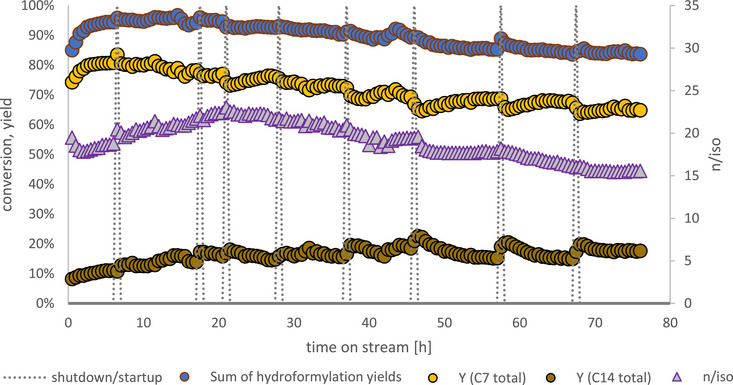
Hexene conversion, product yields and n/iso ratio for the OSN‐assisted reaction system. Reaction conditions: T = 125°C, p = 50 bar, c_0, Rh(acac)(CO)2, reactor_ = 1,44 g L^−1^, c_0, Sulfoxantphos, reactor_ = 8,77 g L^−1^, x_Rh/p_ = 1:4, $\overset{.}{\underset{CO}{n}}\;\;$ = $\overset{.}{\underset{H2\;}{n}}\;$ = 1 mol h^−1^, $\overset{.}{\underset{Hexene}{V}}$ = 16 mL h^−1^, $\overset{.}{\underset{Recycle}{V}}$   =  200 mL h^−1^, $\;\;\overset{.}{\underset{Membrane\;crossflow}{m}}=$ 60 kg h^−1^ Startup procedures and its effects on fluctuating C_7_ and C_14_ yields are highlighted in the SI (Figure S1)

The OSN‐assisted reaction was run in the same setup as the multiphasic one, with the exception of a crossflow cycle added between buffer tank and product container (variant B). Continuous operation was maintained for a total of 76 h while only decreasing to 83% in terms of hexene conversion. Figure 8 shows the results.

While being run for 76 h and so 40 h longer than the multiphasic reaction system, the OSN‐assisted process proved remarkably stable in comparison to the former. Although some aldol condensation was observed due to uncatalyzed, thermal activation at reaction temperature, the reaction mixture remained monophasic throughout the experiment. Spikes in aldol condensate (C_14_) yield occur during overnight standstill, when during cooldown aldol condensation of heptanal (C_7_) continued slightly while hydroformylation stopped earlier without stirred introduction of syngas. A more detailed description of this phenomenon can be found in the SI (Figure S1).

Initially hydroformylation yields increase steeply from 85% to 95%, indicating conversion of the additional prefilled substrate in the membrane loop, which led to less conversion after the startup phase compared to the multiphasic reaction run. The n/iso ratio remains in a range between 15 and 23, therefore indicating the expected high favorability of n‐heptanal over its iso counterpart with a bidentate ligand like Sulfoxantphos, leaving only between 4 and 6% of produced aldehyde to be iso‐heptanal.

After 76 h of continuous runtime, more than twice the amount of the multiphasic experiment, the experiment was stopped, with hydroformylation yields at 83% still considerably above those of the multiphasic experiment after 36 h (72%).

### 2.4 Catalyst Leaching Comparison

**FIGURE 9:**
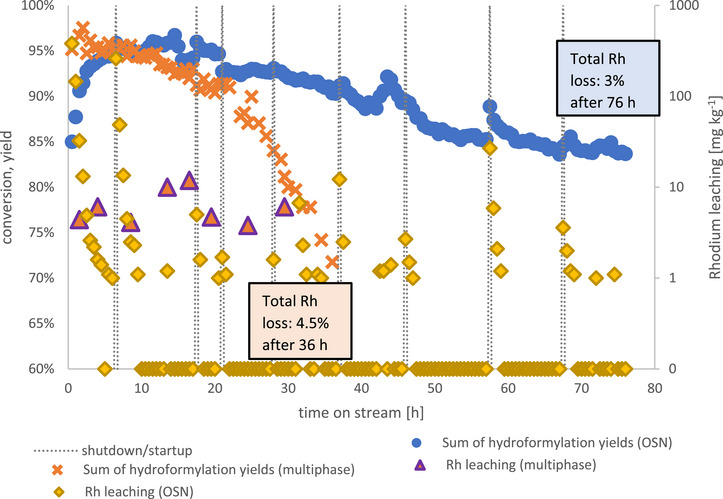
Rhodium leaching and hexene conversion of multiphasic and OSN approach compared Reaction conditions: T = 125°C, p = 50 bar, c_0, Rh(acac)(CO)2, reactor_ = 1,44 g L^−1^, c_0, Sulfoxantphos, reactor_ = 8,77 g L^−1^, x_Rh/p_ = 1:4, $\overset{.}{\underset{CO}{n}}\;\;$ = $\overset{.}{\underset{H2\;}{n}}\;$ = 1 mol h^−1^, $\overset{.}{\underset{Hexene}{V}}$ = 16 mL h^−1^, $\overset{.}{\underset{Recycle}{V}}$   =  200 mL h^−1^, $\;\overset{.}{\underset{Membrane\;crossflow}{m}}=$ 60 kg h^−1^ Sum of hydroformylation yields includes all heptanal produced, including the amount undergoing the intended follow‐up aldol condensation and therefore appearing as aldol product in the product.

Next to product output, continuous processes need to be compared in terms of catalyst loss to assess performance. Rhodium leaching occurs on a higher level in the multiphasic process. With contents in the product ranging between 4 and 12 mg kg^−1^ it generally ranges well above most of the leaching values acquired for the OSN system, which for most time of the experiment detected Rhodium content of the permeate to be below the detection limit of 0.1 mg kg^−1^ (Figure 9). Outliers occur during startup of the continuous experiment, when the membrane proved susceptible to damage after prolonged contact with high concentrations of unreacted substrate. Lesser outliers after the 10 h mark is mostly due to overnight shutdowns of the miniplant, which grant catalyst complexes time to diffuse through the membrane without applied cross‐flow of feed medium. Those are washed out by fresh permeate after continuation of the reaction on the following day, leading to Rhodium content returning quickly to values below the detection limit (Figure 9). Additional information on influences of daily startup procedures on catalyst retention can be found in the SI.

During the entire experiment runtime of 36 h, approx. 4.5% of the initially weighed in Rhodium were lost to the product stream in the multiphasic process. The OSN process lost 3% in 76 h in total. Extrapolating the multiphasic system's loss to a fictive 76 h runtime, the Rh loss would amount to 9.5% and thus be more than thrice as high as for the OSN system during its 76 h on stream. Thus, the OSN process is proven to be significantly more catalyst‐saving. Taking only the 52 h of stable, continuous operation where Rhodium content in the product stream is below the detection limit of 0.1 mg kg^−1^ into account and excluding leaching‐prone startup phases, Rhodium loss would only be less than 0.032 % of the initially weighed in amount, showing a catalyst recycling capability suitable for large‐scale industrial processes, where this loss rate would translate to less than 5% of the weighed in Rhodium being lost to the product during yearly operation of 8000 h.

Both processes, the multiphasic as well as the OSN‐assisted one, suffer from loss of activity during continuous runtime. During the first 3 h continuous operation of the OSN process, observed hydroformylation yield increases. This is due to the additional substrate in the membrane circuit which results in longer time needed to convert all starting material and reach a steady state. From there on, the following 10 h of continuous operation of both processes feature a similar yield of approximately 95 %, as would be expected for both processes having the same amount of Rhodium in the reactor. Mass transport of substrate and product across the liquid‐liquid interface of the multiphasic system seems to be no limiting factor, as no difference in reactivity between mono‐ and multiphasic reaction systems is apparent. After that period of nearly stable operation, observed yields decline in both cases, but steeper in case of the multiphasic system. While another 26 h of continuous operation caused hexene conversion to drop to below 72%, the OSN systems still boasted 90% at that stage. This difference can be explained by the differing catalyst retention properties of the two recycling methods, leading to less active Rhodium sites being available for hydroformylation in the multiphasic system over time. While this is likely not the only source of observed reaction activity loss, as a loss of 4.5% of the initially added Rhodium concurs with ca. 24% less activity after 36 h of continuous runtime, it likely affects other catalyst deactivation mechanisms by concentrating their effects on less Rhodium sites available in the system, thus removing more active Rhodium sites from the reaction. A similar connection can be seen with the multiphasic system, where 3% Rhodium loss concurs with approx. 10% activity loss after 76 h of continuous operation.

## 3 Conclusion

For the first time, this study intensively examined two catalyst recycling strategies next to each other on a single reaction system. After systematically comparing the multiphasic approach with OSN, a clear favorability of OSN became apparent in terms of catalyst recycling ability and activity conservation. As the additional phase boundary layer itself does not impede the multiphasic system in terms of reactivity, as the comparable initial yields of continuous operation show, the high catalyst leaching, over time, renders it inferior to the OSN approach. After only 36 h of continuous runtime the hexene conversion is significantly down by 24%, compared to only 10% with OSN for catalyst recycling after 76 h. With literature frequently reporting catalyst metal leaching to the product in multiphasic systems being in the 1‐digit ppm range [39, 40, 41, 42], alike observed in this work, it is likely close to the limit of recycling capability of this process alternative. Taking into account Rhodium prices of around 143€ g^−1^ and resale prices of approx. 200€ per A4 flat sheet of *oNF‐2* membrane in November 2025 [43, 44], the additional Rhodium refeed needed to compensate the loss during hypothetical 8000 h runtime of the multiphasic process (10 times the starting amount, 95€ in pure Rhodium cost) would be equivalent in cost to 5.75 substitutions of the membrane area during 8000 h of hypothetical runtime of the continuous OSN process with an extrapolated Rhodium loss worth 1.5€. Therefore, a membrane lifetime of 1400 h or more under process conditions would suffice for a cost‐saving applicability of the OSN process alternative over the multiphasic approach with the model reaction system used in this work.

In addition to that, multiphasic catalyst recycling necessarily requires the existence of a suitable, additional solvent that dissolves the catalyst, while at the same time not mixing with the product to form a separate phase. This requirement further limits the applicability of this recycling strategy.

As could be shown, recent commercially available OSN membranes allow for comparable or even more robust processes, whenever a suitable membrane can be found for the reaction system in question. Therefore, the developments in membrane technology have contributed to a greater variety of catalyst recycling options, increasing degrees of freedom for tuning of reaction systems and compositions, as compared to the multiphasic catalyst recycling option, which is much less flexible and bound to maintain the composition of the reaction mixture within narrow operation windows to not lose its biphasicity and thus its recycling capability. Additionally, not all reaction systems offer the possibility to introduce a second liquid phase in the first place.

Although this comparison is based on one specific example, it illustrates the significant challenges involved in developing these two recycling strategies. For extraction by a second phase, compromises in solubility must be made to overcome mass transfer limitations and the reactivity of the system. For membrane‐based systems, this compromise lies in membrane compatibility with the reaction, which implies concentration windows for good separation and stability against by‐products and temperature.

## 4 Experimental Section

### Reaction Setups

For both process options, slightly different apparative setups were needed. While the 250 mL *Parr Instruments* stirred tank reactor and substrate feed instruments (*Shimadzu LC‐10* HPLC pump for hexene, *Bronkhorst Cori‐Flow* flow controllers for hydrogen and carbon monoxide) can be the same, downstream separation units needed to be adjusted. For the multiphasic system, a dip tube removed excessive liquid volume from the reactor to settle into two phases in the decanter. The bottom outlet of it connects to a recycle pump (*FluSys*) to refeed polar catalyst phase back to the reactor at 200 mL h^−^^1^, whereas the lighter, organic product phase is led into a product container with the excess syngas through a back pressure regulator, maintaining constant reaction pressure. For the OSN‐assisted process the downstream tank connected to the reactor dip tube is reassigned to a role as buffer tank for the added secondary membrane separation circuit, as it was no longer needed for settling of a biphasic liquid mixture. Therefore it leveled out the fluctuations in reactor liquid level that would occur if the 60 kg h^−1^ membrane cross‐flow would directly be retrieved from the reactor. To maintain a homogenization of concentrations in buffer tank and reactor, a constant recycling stream of 200 mL h^−1^ was maintained between them via a recycle pump.

A flat sheet membrane module of the *Evonik METcell* series with 52 cm^2^ active membrane area was used to hold the membrane. Permeate passes into a product container through a flowmeter, measuring permeate flow. As the membrane area, based on the membrane screening flux results, was large enough to allow for permeate flow larger than needed to withdraw the same volume that was fed, excess permeate retrieved in the product container was fed back into the reactor via a permeate recycle pump controlled by an additional *Bronkhorst Cori‐Flow* flow controller. That way, constant liquid levels in the setup were ensured, at the same time maintaining set mean residence times. Avoiding overly excessive buffering effects of this recycle on observable product yields, the product container was replaced daily and the permeate fraction stored separately. The product container was kept under a slight syngas pressure to avoid air contamination.

With PEG retention of only 55% (membrane screening results), significant amounts of PEG are lost to the product container each day of continuous operation. Therefore, the removed product fraction was analyzed for its PEG content daily, and a corresponding amount of makeup PEG was introduced. Thus PEG content of the reaction system was ensured to stay within a robust operating window, ensuring Sulfoxantphos to not precipitate.

Both setups were started up in batch mode after filling with the respective amount of starting materials and substrate and run for 6 h to convert the prefilled substrate to a degree comparable with the expected hexene conversion at the start of the continuous operation. After that startup time, the 1‐hexene feed was activated for both reaction systems and continuous operation started. Throughout its runtime, samples were taken regularly from the product outlet stream before entering the product container.

Batch experiments were conducted in 20 mL stainless steel high‐pressure autoclaves with glass inserts. All chemicals were degassed before use and handled using Schlenk techniques or a glovebox.

### Analytics

Yields were determined by GC. Gas chromatography measurements were performed on Shimadzu Nexis GC‐2030 chromatographs equipped with FID and TCD detectors and CP Wax 52 CB and RTX‐1 columns. Samples were prepared by diluting 0.1 mL of product solution with 1 mL of heptane or 1 mL of isopropanol, respectively. Response factors for all compounds were determined by calibration or estimated using the Sternberg effective carbon method [45]. ICP‐MS measurements were conducted using a Shimadzu ICPMS‐2030. Digestions were carried out with a CEM Corp. Mars 6. XRF analysis was performed using a Spectro Xepos C. Sulfoxantphos retention was calculated by subtracting the ratio of Phosphorus concentration in permeate and rententate from 100%.

### Chemicals

1‐hexene (97%) and polyethylene glycol (PEG  200, average molecular weight = 200 g mol^−1^) were purchased from Sigma‐Aldrich, cesium carbonate (99.5%) and [Rh(acac)(CO)_2_] (98.5%) were obtained from Acros Organics. Sodium 4,5‐bis(diphenylphosphino)‐9,9‐dimethyl‐9H‐xanthene‐2,7‐disulfonate (Sulfoxantphos, 99+%) was purchased from AmBeed. Carbon monoxide (99.997%) and hydrogen (99.999%) were obtained from Westfalen AG and Air Liquide.

## Conflicts of Interest

The authors declare no conflict of interest.

## Supporting information




**Supporting File**: chem70609‐sup‐0001‐SuppMat.docx
