# Supplementary material for: Systematic Comparison of Homogeneous Catalyst Recycling Strategies: Organic Solvent Nanofiltration vs. Liquid‐Liquid‐Multiphase
Source: Chemistry. 2025 Dec 24;32(6):e03075. doi: 10.1002/chem.202503075 (PMC12887621; doi:10.1002/chem.202503075)
Supplement: Supplementary file 1 — Supporting File: chem70609‐sup‐0001‐SuppMat.docx [file CHEM-32-e03075-s001.docx]

**Systematic comparison of homogeneous catalyst recycling strategies:
Organic Solvent Nanofiltration vs. Liquid-liquid Multiphase**

S. Störtte ^a^, L. Steinwachs ^a^, R. S. Medhekar ^a^, R. Novemen ^a^, Andreas J. Vorholt ^a,*^

a. Max Planck Institute for Chemical Energy Conversion, Stiftstraße 34-36, 45470 Mülheim an der Ruhr, Germany.

*. Corresponding author

Containing:

4 Tables
3 Figures
6 Pages

Contents

[1 Catalyst retention and standstill phenomena in the OSN-assisted process 2](#_Toc215289608)

[2 CSTR setup for continuous experiments 4](#_Toc215289609)

[3 Membrane testing setup 5](#_Toc215289610)

[4 Membrane test results 5](#_Toc215289611)

[5 Analysis conditions 6](#_Toc215289612)

# Catalyst retention and standstill phenomena in the OSN-assisted process

Fig. S1: Rhodium retention, yields of C_7_ and C_14_ products, and standstills during continuous operation Reaction conditions: T = 125 °C, p = 50 bar, c_0, Rh(acac)(CO)2, reactor_= 1,44 g L^-1^, c_0, Sulfoxantphos, reactor_= 8,77 g L^-1^, x_Rh/P_ = 1:4, $\dot{n}_{CO}$=$\dot{n}_{H2}$= 1 mol h^-1^, $\dot{V}_{Hexene}$= 16 ml h^-1^, $\dot{V}_{Recycle}$ = 200 ml h^‑1^,$\dot{m}_{Membrane crossflow}=$ 60 kg h^-1^

As seen in Figure S1, the Rhodium retention exhibited by the membrane ranges above 99.99% for most of the time during the continuous experiment. Notable exceptions are the two instances of membrane rupture (fault 1) and displacement (fault 2), when a side stream could enter the permeate compartment without traversing the active membrane layer, contaminating the permeate with catalyst from the feed compartment. In both cases the initial separation performance could be reinstated by installing a new cupon from a fresh flat sheet. After that, it took approximately 2 h for the Rhodium contamination in the permeate line to wash out and observed retention to return to values above 99%.

Besides those mechanical impairments, also the cases of overnight standstill caused temporary diminution of catalyst retention. With membrane crossflow being deactivated while the plant is shut down, the Rhodium complexes remaining in the feed compartment were left to their thermic equilibrium, diffusing through the active membrane layer and accumulating in the permeate compartment to some degree, only to be washed out after startup of the miniplant the next working day, when original separation performance is restored by turning on the crossflow. Distinctively, in that regard, is the decrease in observed retention to 96.31% following the standstill after 57.5 h on stream. There, the miniplant was left dormant for a weekend, resulting in larger amounts of Rhodium diffusing across the membrane, compared to all the other standstills that lasted for a single night only.

In addition to those effects on separation performance, the nightly shutdown procedures also caused the alternating, step-wise exchange of C_7_ and C_14_ yields visible in the data. Naturally, after stopping the stirrer, hydroformylation halts quickly due to depletion of available syngas that is no longer introduced by other means than slow diffusion via the surface layer on top of the liquid phase. The side reaction of thermic aldol condensation, on the other hand, is not limited by these factors and continues well into the cooling phase, until reactor temperatures decrease below levels necessary to sustain significant reaction rates. Therefore, with each shutdown sequence, heptanal is converted to aldol products, causing the measured values after next day’s startup to increase step-wise in case of C_14_ products, and decrease in a similar fashion for C_7_ products. A few hours into the continuous operation, these disturbances are leveled out again by newly-formed product reinstating the original ratio of C_7_ and C_14_ products the continuous reaction generates.

# CSTR setup for continuous experiments


Fig. S2: Setup for continuous hydroformylation with multiphasic or OSN-based catalyst recycling option

Table S1: reaction conditions

| Reaction parameters | |
| --- | --- |
| Pressure | 50 bar |
| Reaction temperature | 125°C |
| Stir rate | 2000 rpm |
| CO/H_2_-ratio | 1:1 |
| Catalyst precursor | Rh(acac)(CO)_2_ |
| Ligand | Sulfoxantphos |
| Ligand/metal ratio | 2:1 |
| Brønsted base catalyst (multiphase only) | cesium carbonate |
| Solvent | Polyethyleneglycol-200 (PEG 200) |
| Solvent volume fraction in reactor | 0.3 |
| Substrate feed (1-hexene) | 16 ml h^-1^ |
| CO feed | 1 mol h^-1^ |
| H_2_ feed | 1 mol h^-1^ |
| Rhodium precursor concentration (reactor) | 1.44 g L^-1^ |
| Ligand concentration (reactor) | 8.77 g L^-1^ |
| Brønsted base concentration (reactor, multiphase only) | 45.2 g L^-1^ |
| Rhodium molar loading (per solvent) | 0.3 mol% |
| Ligand molar loading (per solvent) | 0.6 mol% |
| Brønsted base molar loading (per solvent, multiphase only) | 7.5 mol% |

# Membrane testing setup


Fig. S3: Setup for membrane testing in continuous operation with constant feed conditions.
Separation conditions: p=50 bar, c_0, Sulfoxantphos_= 8,77 g L^-1^, substrate-product volume fraction= 0.7, PEG volume fraction=0.3, hexene:heptanal ratio=20:80, $\dot{m}_{Membrane crossflow}=$ 60 kg h^-1^, A_membrane_=52 cm^2^

# Membrane test results

Table S2: Sulfoxantphos and PEG retention, permeate flux of 5 commercially available membranes. Separation conditions: p=50 bar, c_0, Sulfoxantphos_= 8,77 g L^-1^, substrate-product volume fraction= 0.7, PEG volume fraction=0.3, hexene:heptanal ratio=20:80, $\dot{m}_{Membrane crossflow}=$ 60 kg h^-1^, A_membrane_=52 cm^2^Retention was calculated by subtracting the ratio of concentration in permeate and retentate from 100%.

| Table entry | Membrane | Permeate flux [kg m^-2^ h^-1^] | Sulfoxantphos retention [%] | PEG retention [%] |
| --- | --- | --- | --- | --- |
| 1 | Borsig oNF-2 | 2.88 | 98.98 | 55.19 |
| 2 | Borsig oNF-1 | 0.13 | 16.70 | 11.04 |
| 3 | Dow NF 90 | 0.07 | 8.20 | 7.06 |
| 4 | AMS S-3011 | 0.08 | 14.62 | 2.10 |
| 5 | AMS S-3012 | 0.04 | not tested^1^ | 2.85 |

^1^: due to the low permeate flux, not enough sample volume to run XRF analysis could be acquired.

# Analysis conditions

CP-wax CB column (GC, *Shimadzu Nexis GC-2030*)

Table S3: Temperature program used for the CP-wax column. Analysis conditions: Carrier Gas: Helium, p= 251,9 kPa, Linear velocity= 35.0 cm s^-1^, Purge Flow= 3.0 ml min^-1^, Split Ratio= 20.0, V_Injection_= 0.5 µL, T_Injector_=250 °C, T_FID_= 250 °C.

| Table entry (phase) | Start temperature [°C] | End temperature [°C] | Phase duration [min] | Mode | Heating rate [°C min^-1^] |
| --- | --- | --- | --- | --- | --- |
| 1 | 60 | 60 | 3.00 | Hold | 0 |
| 2 | 60 | 250 | 15.83 | Heat | 12 |
| 3 | 250 | 250 | 33.00 | Hold | 0 |

RTX-1 column (GC, *Shimadzu Nexis GC-2030*)

Table S4: Temperature program used for the RTX-1 column. Analysis conditions: Carrier Gas: Helium, p= 255,2 kPa, Linear velocity= 35.0 cm s^-1^, Purge Flow= 3.0 ml min^-1^, Split Ratio= 5.0, V_Injection_= 0.1 µL, T_Injector_=250 °C, T_FID_= 325 °C, T_TCD_= 250 °C.

| Table entry (phase) | Start temperature [°C] | End temperature [°C] | Phase duration [min] | Mode | Heating rate [°C min^-1^] |
| --- | --- | --- | --- | --- | --- |
| 1 | 60 | 60 | 2.00 | Hold | 0 |
| 2 | 60 | 245 | 9.25 | Heat | 20 |
| 3 | 245 | 245 | 20.00 | Hold | 0 |

Digestion procedure for ICP-MS

Samples were prepared by digesting 0.1 g of sample in 20 ml of 1 molar nitric acid solution in a microwave digestion device (*CEM Corp. Mars 6*). The resulting solution was fed into ICP-MS (*Shimadzu ICPMS-2030*) calibrated with external standards using the same method. The result was calculated by dividing the measured Rhodium content of the digested solution by the weighed in amount of original sample.

Digestion procedure for XRF

No digestion procedure was used. Samples were measured directly, since XRF (*Spectro Xepos C*) does not require any input other than the sample and the specification of the amount weighed in.
